# Effect of Metal-Ligand Coordination Complexes on Molecular Dynamics and Structure of Cross-Linked Poly(dimethylosiloxane)

**DOI:** 10.3390/polym12081680

**Published:** 2020-07-28

**Authors:** Angelika Wrzesińska, Izabela Bobowska, Paulina Maczugowska, Joanna Małolepsza, Katarzyna M. Błażewska, Aleksandra Wypych-Puszkarz

**Affiliations:** 1 Department of Molecular Physics, Faculty of Chemistry, Lodz University of Technology, Zeromskiego 116, 90-924 Lodz, Poland; izabela.bobowska@p.lodz.pl (I.B.); paulina.maczugowska@edu.p.lodz.pl (P.M.); 2 Institute of Organic Chemistry, Faculty of Chemistry, Lodz University of Technology, Zeromskiego 116, 90-924 Lodz, Poland; gmach.joanna@gmail.com (J.M.); katarzyna.blazewska@p.lodz.pl (K.M.B.)

**Keywords:** PDMS metal-ligand complexes, molecular dynamics, broadband dielectric spectroscopy, glass transition, fragility index, constrained amorphous phase

## Abstract

Poly(dimethylosiloxane) (PDMS) cross-linked by metal-ligand coordination has a potential functionality for electronic devices applications. In this work, the molecular dynamics of bipyridine (bpy)–PDMS-MeCl_2_ (Me: Mn^2+^, Fe^2+^, Ni^2+^, and Zn^2+^) are investigated by means of broadband dielectric spectroscopy and supported by differential scanning calorimetry and density functional theory calculations. The study of molecular motions covered a broad range of temperatures and frequencies and was performed for the first time for metal-ligand cross-linked PDMS. It was found that the incorporation of bpy moieties into PDMS chain prevents its crystallization. The dielectric permittivity of studied organometallic systems was elevated and almost two times higher (*ε*′ ~4 at 1 MHz) than in neat PDMS. BpyPDMS-MeCl_2_ complexes exhibit slightly higher glass transition temperature and fragility as compared to a neat PDMS. Two segmental type relaxations (*α* and *α_ac_*) were observed in dielectric studies, and their origin was discussed in relation to the molecular structure of investigated complexes. The *α_ac_* relaxation was observed for the first time in amorphous metal-ligand complexes. It originates from the lower mobility of PDMS polymer chains, which are immobilized by metal-ligand coordination centers via bipyridine moieties.

## 1. Introduction

Nowadays, flexible organic electronics hold a great potential in “next generation” applications, such as foldaway displays [1,2], wearable printable electronics [3,4,5], advanced biomedical sensors [3,6,7], communication technology [8,9], and virtual reality devices. Its unique combination of properties, such as flexibility, light weight, potential biodegradability, elasticity, and ductility with a possibility of production by means of cheap, solution-based techniques like spin-coating and printing, generated the tremendous attention of scientist and engineer communities [1,2,3,4,5,6,7,8,9,10].

Currently, the fast-growing market of organic electronics requires constant development of new elastomeric materials with an elevated value of dielectric permittivity (*ε*′) [11]. Chemically or physically cross-linked polymers, which possess a low value of glass transition temperature (*T_g_*) and reversible deformation [12] belong to this type of materials. One of the most popular elastomers commonly used in this area is cross-linked poly(dimethylosiloxane) (PDMS). Moreover, PDMS is characterized by the lowest *T_g_* ~153 K among polymers, is not expensive and susceptible to chemical modification [13]. Numerous strategies of increasing *ε*′ of PDMS have been reported. Ouyang et al. [14] proposed the addition of inorganic nanoparticles with high *ε*′ into the PDMS matrix. A nanocomposite with optimized electro-mechanical properties (***ε*′** ~4 at 100 Hz) was obtained by adding 5 wt% TiO_2_ nanoparticles. Similar methodology was described by Bele et al. [15], where siloxane composites based on polydimethylsiloxane-*α,ω*-diols with different chain lengths as matrices and BaTiO_3_ particles (1, 2, and 5 wt%) as filler were prepared and crosslinked to obtain electroactive elastomeric composite films. The maximum dielectric permittivity value was 4.41 at 10 Hz, for the filler content of 5 wt%. The described approach efficiently raises the value of *ε*′, but at the same time, leads to lower stretchability and higher roughness of the material [15]. Dünki et al. [11] described a series of novel polysiloxanes obtained by introducing nitrile pendant polar groups. The dielectric properties of these materials can be fine-tuned using different proportions of polar nitrile groups, because of their large dipole moment. Due to high dielectric permittivity (up to 18), such polymers are great candidates for dielectric elastomer actuators and flexible electronics.

Currently, very promising organometallic elastomers, cross-linked via noncovalent metal-ligand interactions, are widely explored [16,17,18]. The significant advantage of these materials is the additional polarizability of the metal–ligand bond, which can potentially increase the dielectric permittivity of PDMS. For the flexible electronic devices, it means a reduction in operating voltage and decreased power consumption. Oh et al. [18] reported imidazole modified PDMS cross-linked by zinc-ligand coordination. High *ε*′ (8.30 at 1 MHz) was explained by the dipolar nature of the coordination bond. Recently, Bao et al. [16] introduced Fe(III)-2,6-pyridinedicarboxamide coordination complex into a linear PDMS matrix. The synthesized material possesses the ability to restore a high dielectric strength after recovery from mechanical damage. Later on [17], they described a dielectric elastomer obtained by the incorporation of metal (Fe^2+^ and Zn^2+^)−ligand (bipyridine-5,5-dicarboxylic amide) coordination as cross-linking sites in PDMS. Such materials exhibited increased dielectric permittivity up to 3.5 in comparison to non-modified PDMS (*ε*′ ~2.2).

The use of PDMS cross-linked via noncovalent metal-ligand interactions in advanced technologies, especially in organic electronics, requires a good understanding of the impact of the material structure on its properties. There are only few physical methods, which can provide information about the molecular motions in polymeric materials. Broadband dielectric spectroscopy (BDS) is an excellent tool, which allows a detection of the relaxation processes in a broad temperature and frequency range. A careful analysis of the obtained dielectric spectra can provide information about relaxation processes (their dielectric strength and characteristic relaxation times) in investigated materials [19].

In the case of neat PDMS, there are numerous studies focused on the investigation of its molecular dynamics [20,21,22]. Kirst et al. [20] employed BDS to study linear PDMS of various molecular weights. They observed that the *α*-relaxation shifts towards lower temperatures with decreasing molecular weight, which is in a good accordance with the Fox–Flory model, whereas the relaxation time distribution was found to be independent of length for the linear PDMS chains. They also described an additional slower and broader *α_ac_* relaxation process, which was assigned to the relaxation of the amorphous fraction of PDMS in a close surrounding of crystalline lamellas [20]. Jancelewicz et al. [22] reported that chemical modification by the grafting of alkyl groups into PDMS chain prevents the crystallization process. They also investigated the molecular dynamics of linear PDMS and alkyl-modified PDMS melts, where it was found that segmental dynamics connected with *T_g_* changed linearly in relation to the modifier content increase.

As described above, molecular relaxations of materials depend on many factors—on the one hand, on the chemical composition and structure of the studied systems, which can be controlled by synthesis and processing methods and, on the other hand, on the type and amount of additives, above all, on the specific interactions between different moieties. In the case of functional materials with a complex structure, the key aspect is to define and correctly assign the molecular relaxations and transitions with regard to chemical composition, as well as describe their influence on the operational parameters. According to our best knowledge, only a few studies have been reported for PDMS-based complex materials, and they have concerned an examination of viscoelastic properties (complex modulus), done by dynamic mechanical spectroscopy [23,24].

In the present study, we focused on the dielectric properties and analysis of the molecular dynamics of series PDMS cross-linked by different metal-ligand coordination. BDS, differential scanning calorimetry (DSC), and density functional theory (DFT) calculations have been implemented to understand the relationship between molecular structure and dielectric properties of investigated materials for the first time.

## 2. Materials and Methods

**Figure 1:**
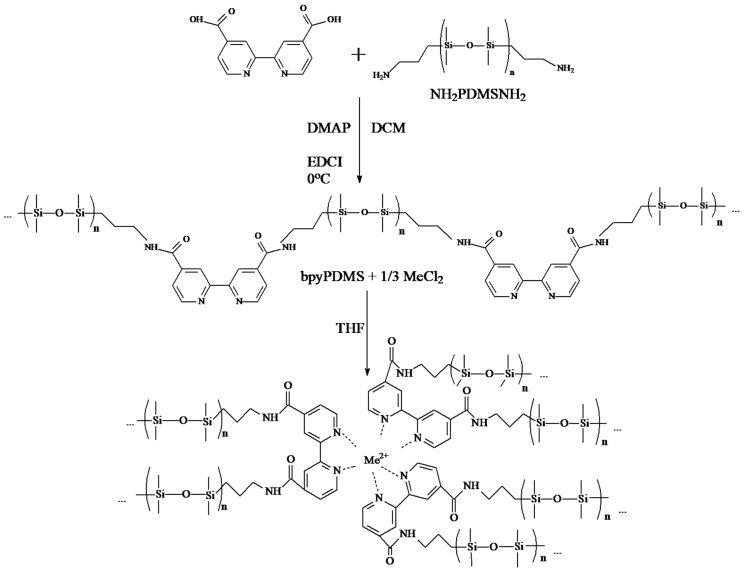
Synthesis scheme and molecular structure of the bpyPDMS-Me(II) system.

The methodology for the synthesis of 2,2′-bipyridine-terminated poly(dimethylsiloxane) from 2,2′-bipyridine-4,4′-dicarboxylic acid 4,4′-dimethyl-2,2′-bipyridine (bpy) and aminopropyl terminated poly(dimethylsiloxane) is illustrated in Figure 1. A recently-reported synthesis route [25] was used. Its modification, described in detail below, included the use of a different ligand moiety (derived from 2,2′-bipyridine-4,4′-dicarboxylic acid) and optimized purification process.

Aminopropyl terminated poly(dimethylsiloxane), referred to later as PDMS (M_n_ = 3350 g/mol, Gelest Inc.) was used for the amide condensation reaction. The reaction was held under nitrogen atmosphere. To the cooled (273 K) mixture of 2,2′-bipyridine-4,4′-dicarboxylic acid (0.100 g, 1.2 eq.), 4-dimethylaminopyridine (0.007 g, 0.2 eq.) dissolved in dry methylene chloride (DCM, 30 mL) and *N*-(3-dimethylaminopropyl)-*N′*-ethylcarbodiimide hydrochloride (EDCI, 0.170 g, 2.5 eq.) dissolved in DCM (20 mL) was added. After 30 min of stirring at 273 K, PDMS (1.07 mL, 1 eq.) dissolved in DCM (30 mL) was added dropwise and stirred for 30 min at 273 K, and then for 15 h at room temperature (~293 K). The resulting solution was diluted with DCM (100 mL) and washed twice with 0.5 M NaOH_aq_ (50 mL), to remove unreacted 2,2′-bipyridine-4,4′-dicarboxylic acid, and with 0.5 M HCl_aq_ (50 mL), to remove side products of EDCI. The organic phase was washed with saturated NaHCO_3aq_ (50 mL) and saturated NaCl_aq_ (50 mL). Next, the organic phase was concentrated under reduced pressure. The resulting polymer was dissolved in a minimal amount of DCM (~0.5 mL) and precipitated upon addition of methanol (2 mL). Yield: 55% (0.6 g, purity ~90%). ^1^H NMR (250 MHz, CDCl_3_, δ/ppm): 8.81 (d, J = 5.2, 2H), 8.66 (bs, 2H), 7.82–7.78 (m, 2H), 6.52 (bs, NH, 2H), 3.53–3.45 (m, 4H), 1.75–1.62 (m, 4H), 0.67–0.56 (m, 4H), 0.07 (bs, 500H). NMR and FTIR spectra of PDMS and obtained product of synthesis (bpyPDMS) are presented in Figures S1–S4 (Supporting Information).

In order to obtain BpyPDMS-MeCl_2_, 0.1 g (2.8 × 10^−5^ M) bpyPDMS was dissolved in 10 mL of toluene and left overnight. Then, 30 μL (9.3 × 10^−6^ M) aliquot of methanol solution containing MeCl_2_ salt (Me: Mn^2+^, Fe^2+^, Ni^2+^, and Zn^2+^, all received from Tokyo Chemical Industry) was subsequently added to the bpyPDMS solution, to obtain PDMS cross-linking by metal-ligand coordination with 1:3 metal:ligand coordination stoichiometry [25].

### 2.1. Density Functional Theory Calculations

Density functional theory (DFT) calculations for [Me(bpy)_3_]^2+^ (Me: Mn^2+^, Fe^2+^, Ni^2+^, Zn^2+^) were performed using the *Gaussian09* program [26]. B3LYP correlation-exchange potential was used for the calculations. It is a hybrid potential containing Lee-Yang-Parr correlation energy functional [27] and Becke’s three-parameter hybrid functional [27]. The 6-31G (d,p) valence double-zeta polarized basis set [28,29] was used to perform geometry optimization. In addition, ECP LanL2DZ pseudopotential [30] was used for transition metal atoms. The natural bonding orbitals (NBO) analysis [31,32] was used to obtain more accurate partial charges.

### 2.2. Differential Scanning Calorimetry (DSC)

DSC thermograms were recorded with the DSC 3 instrument from Mettler Toledo. Samples were heated at a rate of 10 K/min with a 2 mL/min nitrogen gas purge in standard, sealed aluminum crucibles. Measurements were performed in a temperature range from 133 to 373 K, and results of the second heating run were analyzed. The temperature and heat flow were calibrated using indium and zinc melting point standards. Values of *T_g_* were defined as midpoints of respective glass transition step determined by means of STAR^e^ Evaluation Software.

### 2.3. Broadband Dielectric Spectroscopy (BDS)

BDS measurements were performed using a Novocontrol^®^ GmbH Concept 80 Broadband Dielectric Spectrometer. Measurements were performed in a frequency range from 10^−1^ to 10^6^ Hz in the temperature regimes during heating trace 143–373 K. The temperature was controlled using a Novocontrol^®^ Quatro Cryosystem nitrogen cryostat, with a stability better than 0.1°. The analysis of all experimental dielectric data was done using Novocontrol WinFit software. For all measurements of high quality Novocontrol interdigitated electrodes with diameter 15 mm, electrode basic structure size 75 μm, and loss factor tan(δ) ~0.001 were used.

## 3. Results and Discussion

### 3.1. Density Functional Theory Calculations

**Figure 2:**
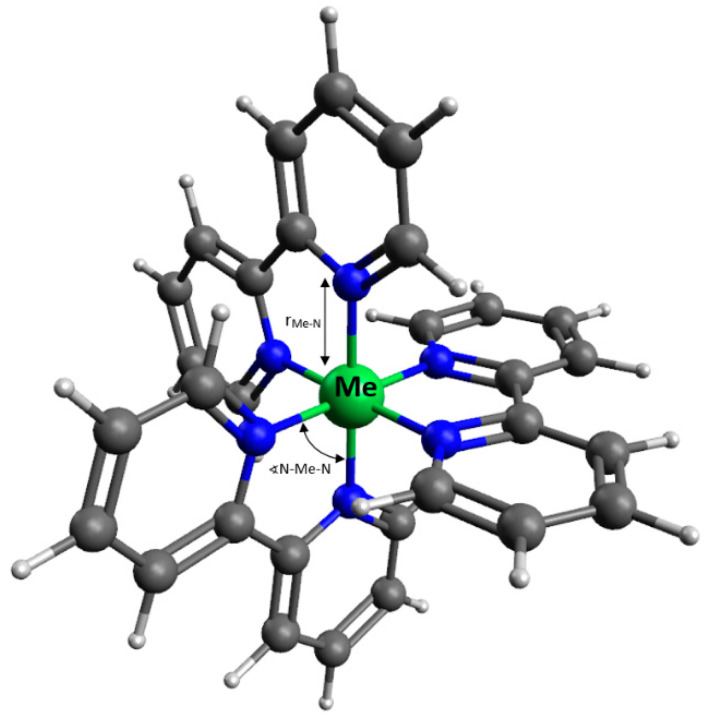
Geometry of optimized Me-bpy coordination complex on the example of bpyPDMS-NiCl_2_.

For the sake of simplicity, the DFT calculations were performed for a single metal-ligand complex of each type. All optimized complexes had octahedron-like geometry (Figure 2, Figure S5 (Supporting Information)).

The total spin in the ground state and the average geometry parameters for each complex used in the calculations are shown in Table 1. Ground-state spins of the complexes were determined based on literature data [33,34]. The values of standard deviations were used to determine the irregularities of the molecule’s geometry Table 2. The detailed geometry-optimized structure of Me-bpy complexes is listed in Table S1 (Supporting Information).

Coordination bond lengths are arranged in the following order: r_Fe-N_ < r_Ni-N_ < r_Zn-N_ < r_Mn-N_. Based on the standard deviation and the ground state parameters, it can be concluded that the bpy-ZnCl_2_ and bpy-MnCl_2_ differ the most from the octahedral structure.

Due to the symmetry of the investigated complexes, the overall dipole moments of each system is close to zero. For this reason, the dipole moment was calculated only for coordination bonds, in order to better reflect the differences between individual complexes. Partial charges were calculated based on the NBO method. None of the created complexes exhibit perfect octahedral organization (see Table 2), where standard deviations $\sigma_{∢}$ [°] exhibit a rate of deviation from perfect assumed symmetry. According to this, contribution to dipole moment created from the complex will be highest for systems with the highest rate of deviation, for Mn and Zn, and the smallest for Ni and Fe.

### 3.2. Differential Scanning Calorimetry (DSC)

**Figure 3:**
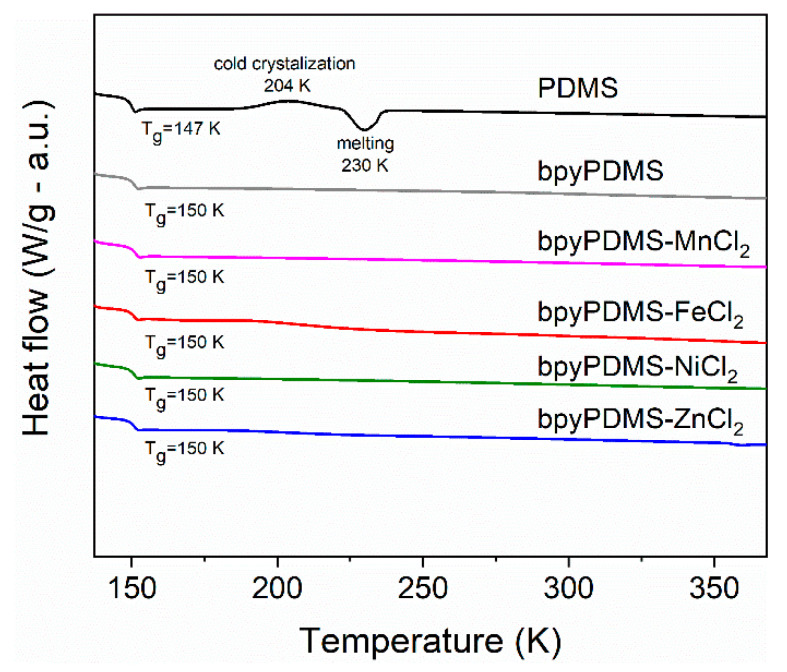
DSC thermograms of PDMS, bpyPDMS, and metalloorganic complexes (thermograms are vertically shifted for better visualization).

Figure 3 shows DSC thermograms obtained from PDMS, bpyPDMS, and bpyPDMS, crosslinked by metal-ligand coordination (organometallics). Measured PDMS is characterized by *T_g_* at 147 K, cold crystallization temperature at 204 K, and melting temperature at 230 K, which is in good agreement with literature data [25].

The bpyPDMS material does not exhibit peaks corresponding to the crystallization and melting of the PDMS chains, due to the fact that the bpy units can act as a spacer and prevent siloxane unit from specific ordering, and thus inhibit crystallization. The *T_g_* value for this material was found to be 150 K, i.e., 3 K higher than in neat PDMS. This may come from the linkage of PDMS chains through bpy that causes the hindering of segmental motions of the chains.

Further analysis of DSC measurements for bpyPDMS-Me(II) materials allowed to state that *T_g_* for all complexes is around 150 K [23], which is similar to bpyPDMS. Therefore, it can be concluded that complexing by using chosen metal ions has no influence on *T_g_* value of final complexes. The obtained materials demonstrated outstanding elasticity above *T_g_*, which is very important in connection with the potential applications of these materials in, for example, organic electronics [17].

### 3.3. Broadband Dielectric Spectroscopy

**Isothermal representation of dielectric response for the studied systems.** The complex dielectric function ε* can be expressed as: ε* = *ε*′ − i*ε*″, where: *ε*′ and *ε*″ are the real and the imaginary (loss) parts of dielectric permittivity, respectively, and i is an imaginary number, being a solution of the equation i^2^ = −1.

**Figure 4:**
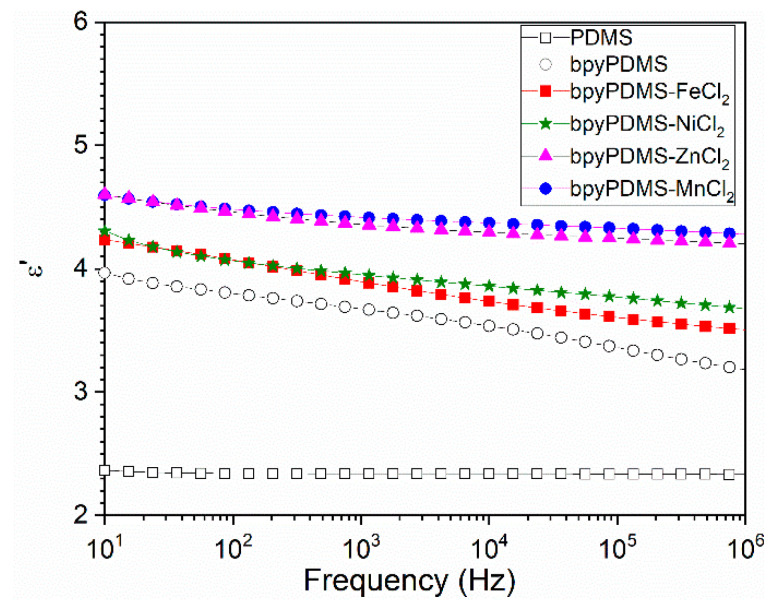
Frequency dependence of real part of dielectric permittivity *ε′* at 293 K of PDMS, and metalloorganic complexes.

Isothermal studies of real-part dielectric permittivity (Figure 4) showed flat response over a broad frequency range. Each of the obtained coordinated systems exhibited a higher value of dielectric permittivity than neat PDMS. Introducing metal-ligand coordination bonds into the polymer networks improved the dielectric properties of material, because of the dipolar nature of the coordination bonds [16,18]. All cross-linked bpy-PDMS systems displayed elevated dielectric permittivity values. The highest value of *ε*′ = 4.3 at 1 MHz was observed for bpyPDMS-ZnCl_2_, which is almost twice as high as neat PDMS. This correlates with the values of dipole moments of coordination bonds obtained in the DFT calculations.

It is commonly known that the value of dielectric permittivity is directly proportional to polarizability, and this comes from the chemical nature of the material. Polarizability (*P*) is given by:

*P* = *N*_0_ <μ> (1)

where *N*_0_ is number of identical molecules having effective dipole moments and the electric dipole moment <μ>:

<μ> = *αE*_loc_ = (*α_e_* + *α_a_* + *α_o_* + *α_ch_*)*E*_loc_ (2)

Dipole moment <μ> is equal to multiplication of polarizability (*α*) and electrical local field (*E*_loc_), where polarizability (*α*) may come from different contributions, like: electronic, atomic, orientation, and space charge, (*α_e_* + *α_a_* + *α_o_* + *α_ch_*), respectively.

As in a homogeneous, linear, non-dispersive and isotropic dielectric medium, the polarization is connected and proportional to the electric field *E* by the formula:

*P* = (*ε*′ – 1) *ε*_0_*E* = χ’ *ε*_0_*E* (3)

where *ε*_0_ is the vacuum permittivity, and χ’ is the real part of electric susceptibility of the medium (in this case simplifies to a scalar) and *E* is an electric field.

Comparing the bpyPDMS system with Me-bpyPDMS, we can assume that the atomic and electron polarization will be unchanged in the mentioned systems. Due to the creation of the metal-ligand complex system, which deviates from perfect octahedral symmetry, thus exhibiting no zero dipole moment, we enhance the contribution of orientation polarization in the investigated materials. Accordingly, the values of *ε*′ for metalloorganic complexes increased in a direct proportion to the theoretically calculated increase of the dipole moment of the metal-ligand bonds (Table 2). Such an observation is in good agreement with the already presented theoretical expectations. In line with Equation (2), we observed higher values of dielectric permittivity as compared to neat PDMS, as well as bpyPDMS. The observed dielectric permittivity for investigated complexes is the highest when standard deviations $\sigma_{∢}$ [^o^] possess the highest value. Frequency dependence of loss tan is presented in Figure S6 (Supporting Information).

**Figure 5:**
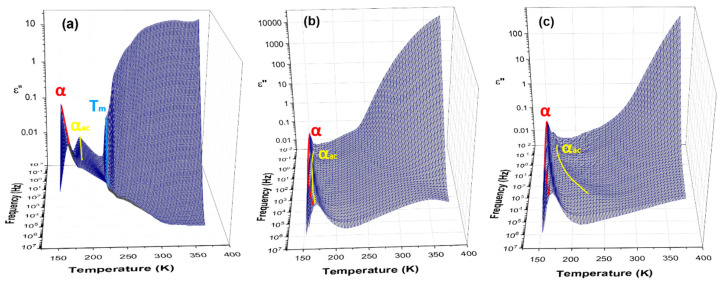
Frequency-temperature dependences of *ε*″ for (**a**) PDMS, (**b**) bpyPDMS, and (**c**) bpyPDMS-ZnCl_2_ samples. The solid lines, drawn as a guide for eyes, indicate the detected relaxation processes *α* and *α_ac_*.

**Temperature Dependence of Dielectric Relaxation.**Figure 5 shows different molecular phenomena that can be detected in the investigated systems upon heating. The representation of the imaginary part of epsilon (*ε*″) has been chosen to reveal the presence of different transitions. PDMS is a well-known type B polymer, where the dipole moment is rigidly attached perpendicular to the chain skeleton, and for that reason only, segmental motions, called *α*-processes, are observed. Starting from the lowest temperature, the first segmental type relaxation in PDMS is assigned as *α*, and at frequency ~1 Hz, it appears at a temperature of 153 K. The second relaxation *α_ac_* is the *α* process in a constrained amorphous phase, which arises at the higher temperature of 164 K at a frequency ~1 Hz. No secondary relaxation processes were detected.

The *α* relaxation can be assigned to the cooperative movements within the main chain of polymer backbone of PDMS, thus can be connected with *T_g_*, whereas *α_ac_* can be associated with lower mobility, i.e., constrained motions of PDMS chains that participate in the creation of crystalline phase [21,35]. At a temperature of about 230 K (Figure 5a), one can observe an increase of imaginary part of dielectric permittivity that results from the melting of crystalline lamellas of PDMS and release of dipoles from crystalline phase. At a higher temperature, the representation of *ε*″ is complex and comprises conductivity phenomena. The analysis of conduction mechanism in PDMS and its metalloorganic complexes at higher temperatures is not a goal of this work, and will be discussed in a further paper.

**Figure 6:**
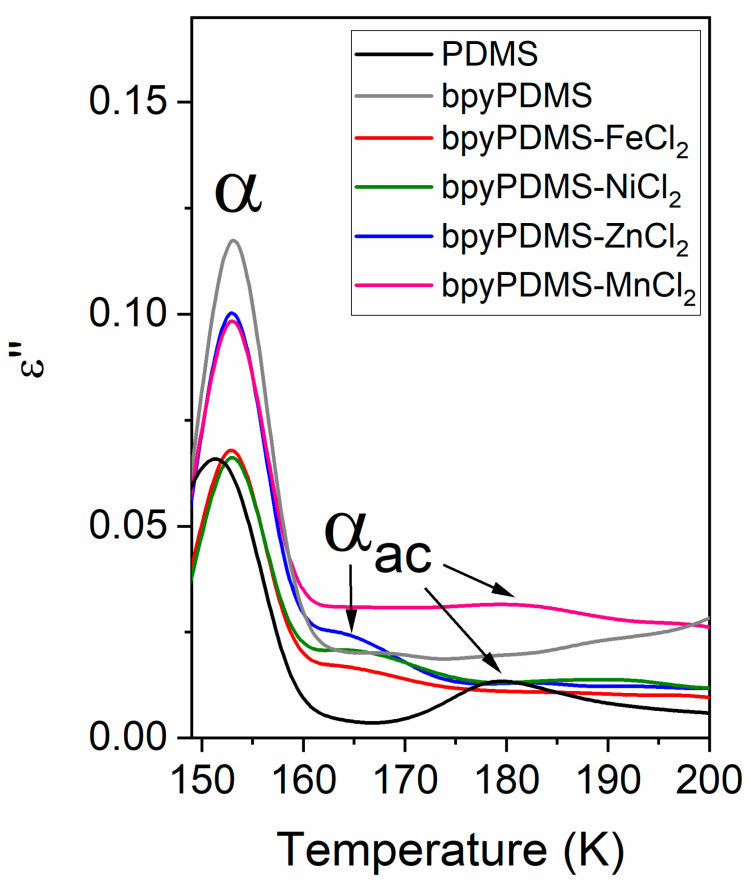
Dielectric loss *ε*″ vs. temperature at frequency 1.15 Hz.

In Figure 6, dielectric loss *ε*″ vs. temperature at frequency 1 Hz plots for different samples are compared. One can observe a shift of *α* relaxation process in bpyPDMS and metal-ligand coordination to a slightly higher temperature, as compared to neat PDMS.

It is important to point out that the maximum *α* relaxation process of bpyPDMS and its complexes is located at a similar position, that is, in good agreement with the DSC results (Figure 3). These findings can be explained by definition of Kuhn segment [36], claiming that PDMS polymer chains return to their original mobility at distance above 5 monomers from the point of immobilization. Thus, immobilization of bpyPDMS chain by metal-ligand complex formation does not significantly affect the maximum position of the *α* relaxation process, but affects dielectric parameters describing this phenomenon.

It should be pointed out that the *α_ac_* relaxation was detected in PDMS and its metalloorganic complexes, but the molecular assignments of this phenomenon in investigated compounds are completely different.

The behavior of the *α_ac_* process in the investigated systems cross-linked by metal-ligand complexes seems to be unusual and very interesting, because it is created in fully amorphous compounds.

We would like to underline that complex formation in investigated systems influences both relaxations, i.e., *α* and *α_ac_*. In the case of *α* relaxation, it is clearly visible by looking at Figure 6, as well as by analyzing the delta epsilon parameters of this process, presented in Table 2. One can notice that the dielectric strength of *α* relaxation increases, along with an increase of the dipole moment of created complexes (see Table 2). The slower *α_ac_* process is characterized by a much lower relaxation strength as compared to *α* relaxation, however, looking at Figure 6, one can notice a similar increase in dielectric strength of *α_ac_* process, which is in agreement with calculated dipole moment, where *α_ac_* amplitude is the highest for Zn-bpy and Mn-bpy complexes. In the case of the Mn-bpy complex, it should be point out that the created structure of this complex is the most deviated from the perfect octahedral symmetry, which is typical for investigated metal-bpy complexes. Based on the standard deviation and the ground state parameters, it can be concluded that bpy-ZnCl_2_ and bpy-MnCl_2_ differ the most from the octahedral structure.

**Figure 7:**
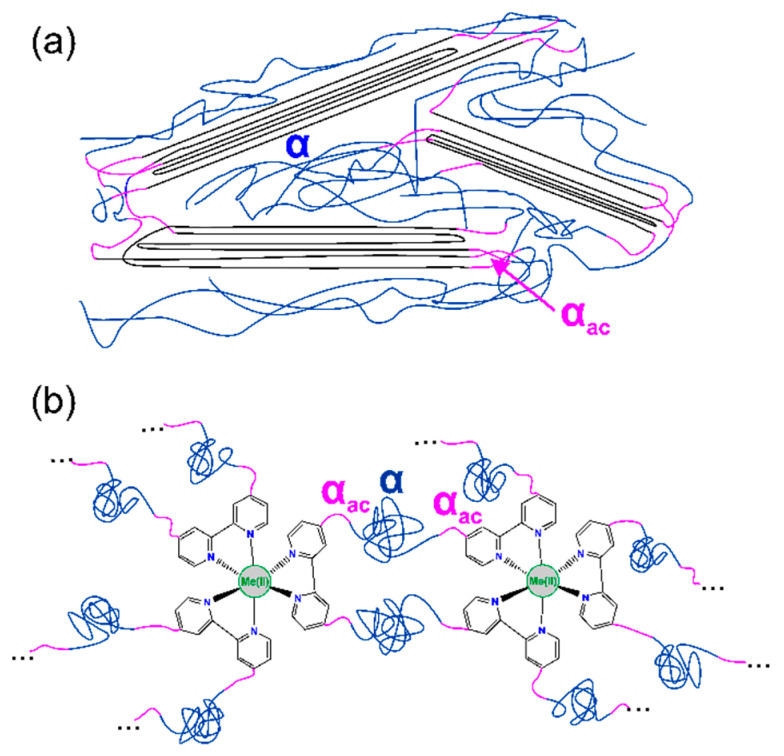
Schematic illustration of origin of *α* and *α_ac_* relaxations in (**a**) non-modified PDMS and (**b**) PDMS cross-linked by metal-ligand coordination (size of the metal-ligand complexes is enlarged to show the structure of the material).

In the case of neat PDMS, the *α_ac_* relaxation comes from the constrained mobility of PDMS chains that participate in creation of crystalline phase, whereas for the PDMS complexes BDS and DSC, measurements evidenced that chemical incorporation of bpy moieties suppresses the crystallization process of PDMS and leads to the creation of fully amorphous systems (see Figure 3). For this reason, it can be supposed that that *α_ac_* relaxation in bpyPDMS and its metal complexes is due to the lower mobility of PDMS polymer chains that are immobilized by bpy moieties. In Figure 7, one can see a schematic illustration of origin for *α* and *α_ac_* relaxations in neat PDMS and its metalloorganic complexes.

In order to examine the influence of metalloorganic complex formation on the segmental relaxation process of PDMS chains, i.e., the *α* relaxation, we analyzed their dielectric response by applying the Havriliak–Negami (HN) equation:

(4) $$\varepsilon^{*\;}=\varepsilon_{\infty }+\frac{\Delta \varepsilon }{{\left(1+{\left(i\omega \tau \right)}^{\alpha_{HN\;}}\right)}^{\beta_{HN}}}\;$$

where ω is an angular frequency, Δε is the dielectric relaxation strength defined as Δε = $\varepsilon_{st}$ $–\;\varepsilon_{\infty }$ ($\varepsilon_{st}$ and $\;\varepsilon_{\infty }$ are the low and the high frequency limits of the real part of the dielectric function, respectively), *τ* is the characteristic relaxation time, whereas $\;\alpha_{HN}$ and $\;\beta_{HN}$ are HN parameters, which correspond to the width and asymmetry of the loss peak, respectively.

**Figure 8:**
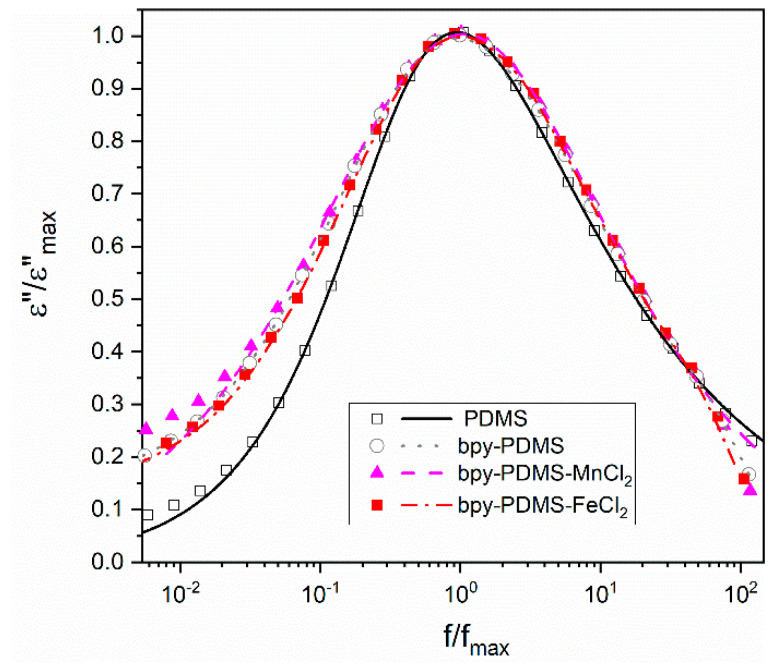
Normalized loss curves for PDMS, bpyPDMS and chosen metalloorganic complexes collected at 163 K. Symbols correspond to experimental points, whereas drawn lines are curves obtained from a WinFit analysis (Figure S7).

Figure 8 shows the normalized representation of *ε*″ (T) collected for PDMS and chosen metalloorganic complexes compounds in order to exhibit influence of bpy incorporation and complex formation on segmental PDMS dynamics. For this reason, different dielectric parameters, like: *τ* (characteristic relaxation time), *τ*_max_ (relaxation time taken from maximum of detected process), dielectric strength, as well as *α_HN_*, *β_HN_* describing width and asymmetry, respectively, were analyzed in Table 3. Mentioned parameters were obtained from fitting of the *α* process at the temperature of 163 K. Figure 8, showed changes in the width and symmetry of these relaxation processes after the incorporation of bpy moieties to PDMS. The temperature 163 K was selected, because at this temperature, the maximum of *α* relaxation is located in the middle of the frequency window that guarantees the proper visibility of low and high frequency shoulders of this process, which is needed for further procedure of the normalization. It is important to point out that the segmental relaxation time of *α* process for PDMS and its complexes is almost unchanged, as revealed in similar relaxation time values (*τ*), whereas *τ*_max_ values (relaxation time calculated from maximum of *ε*″ representation) increase after the incorporation of bpy moieties to PDMS. The incorporation of bpy into PDMS chains apparently slows down the segmental relaxation time, that was evidenced by an increase of *Tg* value (Figure 3) and the shift of *α* relaxation maximum to higher temperatures (Figure 6). One can notice also, that incorporation of bpy moieties into PDMS leads to the broadening of distribution of relaxation time, as reflected in the decrease of *α_HN_* parameter from 0.79 (for PDMS) to ca. 0.6 value for bpyPDMS and its metalloorganic complexes. In turn, more than 30% of the larger value of the *β_HN_* parameter, as obtained for the metalloorganic polymers compared to the neat PDMS, reflects a significant decrease in asymmetry of the *α* process in the investigated systems. In Figure 8, where normalized loss curves for PDMS and chosen complexes are shown, one can observed that bpy presence broadens the distribution and improves the symmetry of *α* relaxation time.

**Figure 9:**
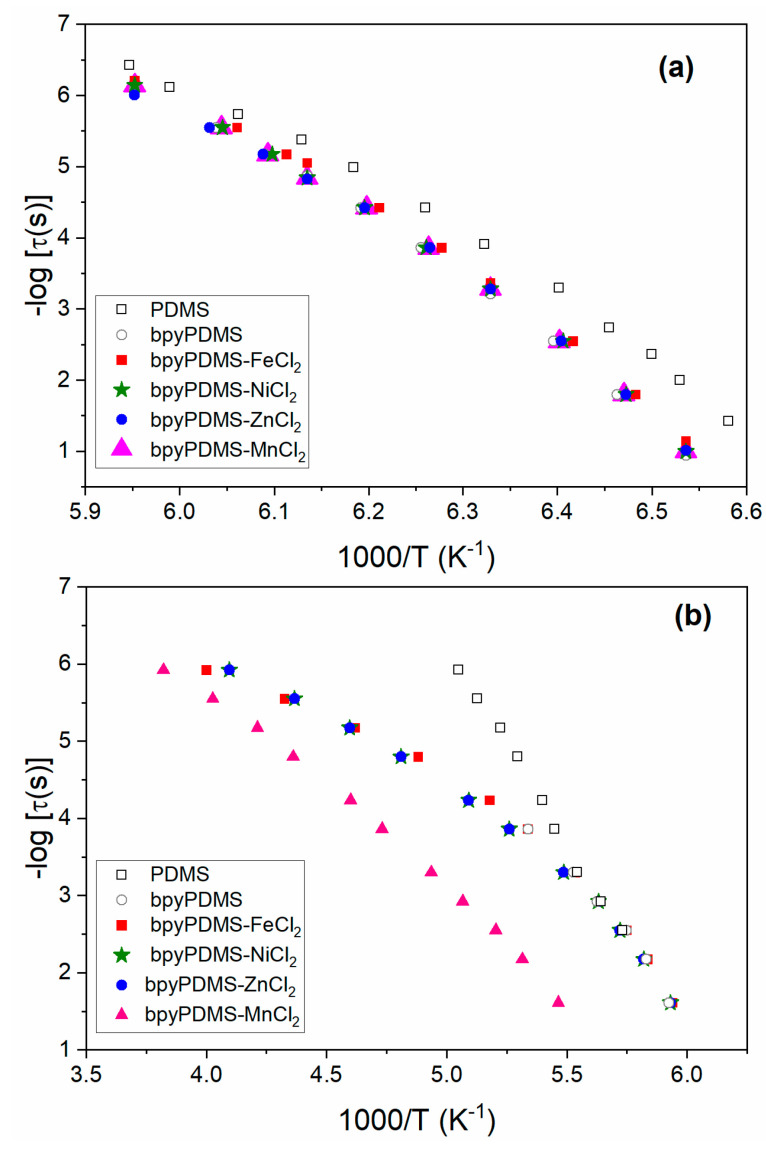
The activation maps for: (**a**) cooperative movements of amorphous chains, i.e., *α* relaxation, (**b**) cooperative motions in constrained phase, i.e., *α_ac_* relaxation for PDMS, bpyPDMS and metalloorganic complexes. The points were taken from the maxima of relaxation processes in *ε*″ representation (Figure 5).

**VFTH analysis–relaxation maps and fragility.** The temperature dependences of detected relaxations for all samples are presented as relaxation maps (Figure 9).

For all these systems, the segmental dynamics (*α* and *α_ac_* relaxations) were interpreted in terms of the Vogel–Fulcher–Tammann–Hesse (VFTH) approach. The obtained temperature dependencies of the segmental relaxation times (Figure 9a,b) were fitted with the VFTH equation [37,38].

(5) $$\tau =\tau_{0\;}\exp\left(\frac{B}{T-T_{0}}\right)$$

where *τ*_0_ and *B* are constants, *T*_0_ is the ideal glass transition or Vogel temperature, usually 30–70 K below *T_g_* [39]. The obtained parameters from fitting VFTH are listed in Table 4.

The *α* process is assigned to the segmental motions, and is sometimes referred to in the literature to the dynamic glass transition temperature *T_g_^DRS^*, which can be calculated using the obtained VFTH parameters, according to the procedure proposed by Saad et al. [19]:

(6) $$T_{g}^{DRS}=T_{ref}=\frac{B}{\ln\left(\frac{1}{\tau_{0}}\right)}+T_{0}$$

where *T_ref_* is the temperature at which the segmental relaxation time *s* is 1 s. It is worth noting that in the literature another practice can also be found e.g., *T_ref_*, at which *τ* = 100 s [40].

In the next step, it is possible to calculate the fragility (*F*), which is a measure of the ability of material to change its conformation across the glass transition region. Thus, the fragility reflexes cooperativity of the system and its correlation with the degree of intermolecular coupling of polymer chains. The relation between VFTH parameters and *F* value [41] is given by:

(7) $$F=\left(\frac{B}{T_{ref}}\right)/\left[\ln\left(10\right)/{\left(1-\frac{T_{0}}{T_{ref}}\right)}^{2}\right]$$

The values of VFTH parameters (*τ*_0_, *B, T*_0_) obtained from the fitting and the calculated *F* values, are collected in Table 4.

The fragility value (*F*) of 88, calculated for linear PDMS, is in accordance with the literature-reported values (85–90) [41,42]. An incorporation of bpy moieties and metal-ligand complexes do not change the fragility of the PDMS chain significantly (see Table 4).

The interpretation of the fragility index in PDMS is rather complex, and a few theoretical works were already proposed. Sokolov et al. [42] reported that an increase in molecular weight does not have a large influence on structural relaxation with regard to the flexibility of the polymeric chain. Furthermore, in the case of PDMS, there is no dependence of fragility on molecular weight [43,44]. Dudowicz et al. [45] suggested that the flexibility of the polymer backbone and side group plays a crucial role in a polymer fragility, because of the different frustration of chain packing for each polymer. PDMS is the highly flexible polymer. This feature is caused by the presence of the Si–O–Si– backbone. Accordingly, it is expected that an increase in the molecular weight of flexible polymer should not significantly modify its properties, for the reason that motions of different parts of the molecules become decoupled on a short distance along the backbone [42]. Our findings are in good agreement with above presented interpretation.

In Figure 9b, an activation maps for cooperative motions in the constrained phase, i.e., *α_ac_* relaxation is shown. One can observe a different trace of neat PDMS *α_ac_* curve as compared to the PDMS metalloorganic complexes. The observed difference comes from the different origin of *α_ac_* relaxation in PDMS and bpyPDMS complexes, as already discussed earlier. In a neat PDMS, *α_ac_* relaxation is connected with the presence of crystalline phase, whereas in the case of bpyPDMS-MeCl_2_ samples, *α_ac_* relaxation exists because of the immobilization of a fragment of the main PDMS chain, which is directly connected to metal-bpy complexes, see Figure 7a,b. We suppose that behavior of *α_ac_* relaxation is affected by coordination bond lengths between metal-bpy and their dipole moments.

In the case of bpyPDMS-MnCl_2_, one can clearly observed the shift of *α_ac_* relaxation to higher temperature (i.e., lower values 1000/T), with an increase of Me-bpy bond lengths and dipole moments of coordinates, as compared to other samples (Figure 9b).

## 4. Conclusions

Through a combination of experimental BDS and DSC methods and DFT theoretical calculations, molecular dynamics in poly(dimethylosiloxane) cross-linked by metal-ligand coordination were investigated. A direct dependence between the real part of dielectric permittivity (*ε*′) and dipole moment of investigated metal-ligand complexes was described for the first time. The highest value of *ε*′ = 4.3 at 1 MHz was reported for bpyPDMS-ZnCl_2_, which is almost twice as high as neat PDMS. The chemical incorporation of bpy moieties significantly suppressed crystallization in investigated PDMS polymeric chains. For all these systems, the segmental motions were interpreted in terms of VFTH approach. The segmental relaxation time of *α* process for PDMS and its complexes is almost unchanged, as revealed in similar relaxation time values (*τ*), whereas *τ*_max_ values increase after incorporation of bpy moieties to PDMS. An observed increase of *T_g_* as seen by DSC measurements is the result of increases in relaxation time distribution in investigated complexes. The relaxation process of amorphous constrained phase (*α_ac_*) in neat PDMS originated from lower mobility of the polymeric chain, connected with the crystalline phase. Relaxation *α_ac_* in systems cross-linked by metal-ligand coordination was described for the first time and assigned to the immobilization of a fragment of PDMS chain by bpy-metal complexes. The fragility index was similar for all investigated samples, which is consistent with no change in *α* relaxation. In summary, the research strategy proposed in this work permits a better understanding of structure-dynamic relationship of novel metal-ligand cross-linked polymers. This approach can be further extended to similar organometallic networks for evaluating their usability in flexible electronic devices.

## Figures and Tables

**Table 1:** The total spins of the complexes in the ground state [33,34].

Complex	Cationic Metal Electronic Structure	Ground State (S)
[Fe(bpy)_3_]^2+^	[Ar]4s^0^3d^6^	0
[Ni(bpy)_3_]^2+^	[Ar]4s^0^3d^8^	2/3
[Zn(bpy)_3_]^2+^	[Ar]4s^0^3d^10^	0
[Mn(bpy)_3_]^2+^	[Ar]4s^0^3d^5^	5/2

**Table 2:** The average length of coordinate bonds r_Me-N_, average values of angles ∢ N–Me–N and dipole moment of coordinate bonds µ_b_ for the optimized structures with values of standard deviations for bonds length $\sigma_{r}$ and angles $\sigma_{∢}$.

	[Fe(bpy)_3_]^2+^	[Zn(bpy)_3_]^2+^	[Ni(bpy)_3_]^2+^	[Mn(bpy)_3_]^2+^
r_Me-N_ [Å]	1.9544	2.2594	2.1407	2.3115
$\sigma_{r}$ [Å]	0.0004	0.0009	0.0006	0.0011
∢ N–Me–N [°]	89.26	87.78	88.51	87.55
$\sigma_{∢}$ [°]	5.33	10.22	8.08	11.36
µ_b_ [D]	3.09	21.05	12.86	19.20

**Table 3:** Havriliak–Negami (HN) parameters for α-relaxation processes in studied PDMS, bpyPDMS, and metalloorganic complexes obtained from the analysis of dielectric spectra at 163 K.

Sample	*τ* (s)	*τ*_max_ (s)	Δ*ε*	*α_HN_*	*β_HN_*
PDMS	1.1 × 10^−5^	4.8 × 10^−6^	0.66	0.79	0.49
bpyPDMS	1.3 × 10^−5^	1.0 × 10^−5^	0.67	0.59	0.85
bpyPDMS-FeCl_2_	9.8 × 10^−6^	7.6 × 10^−6^	0.43	0.60	0.85
bpyPDMS-NiCl_2_	1.1 × 10^−5^	8.9 × 10^−6^	0.40	0.59	0.88
bpyPDMS-ZnCl_2_	1.2 × 10^−5^	9.2 × 10^−6^	0.60	0.59	0.86
bpyPDMS-MnCl_2_	1.1 × 10^−5^	1.0 × 10^−5^	0.54	0.55	0.96

**Table 4:** VFTH and fragility parameters for the segmental relaxation processes of PDMS, bpyPDMS and metalloorganic complexes determined from dielectric studies.

Sample	Ln|*τ*_0_ (s)|	B	*T*_0_ (K)	*T_g_^DRS^* (K)	*F*
PDMS	−30.0 ± 0.7	588 ± 36	129.9 ± 0.8	150.8	88
bpyPDMS	−34.9 ± 0.6	860 ± 38	126.7 ± 0.7	152.5	86
bpyPDMS-FeCl_2_	−34.0 ± 0.9	792 ± 54	127.8 ± 1.0	152.5	86
bpyPDMS-NiCl_2_	−34.8 ± 1.3	854 ±80	126.6 ± 1.5	152.5	85
bpyPDMS-ZnCl_2_	−33.2 ± 1.1	760 ± 65	128.3 ± 1.2	152.5	86
bpyPDMS-MnCl_2_	−34.5 ± 1.0	838 ± 61	126.9 ± 1.1	152.5	85

## Supplementary Materials

The following are available online at https://www.mdpi.com/2073-4360/12/8/1680/s1. Figure S1: ^1^H NMR spectrum of 4,4′-dimethyl-2,2′-bipyridine (bpy) in D_2_O; Figure S2: ^1^H NMR spectrum of aminopropyl terminated poly(dimethylsiloxane) (PDMS) in CDCl_3_; Figure S3: ^1^H NMR spectrum of 2,2′-bipyridine-terminated poly(dimethylsiloxane)(bpyPDMS) in CDCl_3_; Figure S4: FT-IR spectra of bpy, bpyPDMS, and PDMS in the range of 1500–2000 cm^−1^; Figure S5: Geometry of optimized coordination compounds (Me: Mn^2+^, Fe^2+^, Ni^2+^, Zn^2+^); Table S1: Geometry-optimized structure of 2,2′-bipyridine complexes; Figure S6: Frequency dependence of loss tangent at 293 K of PDMS and its metalloorganic complexes; Figure S7: Exemplary of fitting procedure using WinFit software for bpyPDMS-FeCl_2_ metalloorganic complex.
