# Supplementary material for: Effect of Metal-Ligand Coordination Complexes on Molecular Dynamics and Structure of Cross-Linked Poly(dimethylosiloxane)
Source: Polymers (Basel). 2020 Jul 28;12(8):1680. doi: 10.3390/polym12081680 (PMC7465896; doi:10.3390/polym12081680)
Supplement: Supplementary file 1 [file polymers-12-01680-s001.pdf]

Supporting Information

Effect of Metal-Ligand Coordination Complexes on Molecular Dynamics and Structure of Cross-Linked Poly(dimethylosiloxane)

Angelika Wrzesińska [1,*], Izabela Bobowska [1], Paulina Maczugowska [1], Joanna Małolepsza [2], Katarzyna M. Błażewska [2] and Aleksandra Wypych-Puszkarz [1,*]

[1] Department of Molecular Physics, Faculty of Chemistry, Lodz University of Technology, Zeromskiego 116, 90-924 Lodz, Poland; izabela.bobowska@p.lodz.pl (I.B.); paulina.maczugowska@edu.p.lodz.pl (P.M.)

[2] Institute of Organic Chemistry, Faculty of Chemistry, Lodz University of Technology, Zeromskiego 116, 90-924 Lodz, Poland; gmach.joanna@gmail.com (J.M.); katarzyna.blazewska@p.lodz.pl (K.M.B.)

* Correspondence: aleksandra.wypych@p.lodz.pl (A.W.); angelika.wrzesinska@edu.p.lodz.pl (A.W.-P.); Tel.: +48-42-631-32-05 (A.W.-P.)

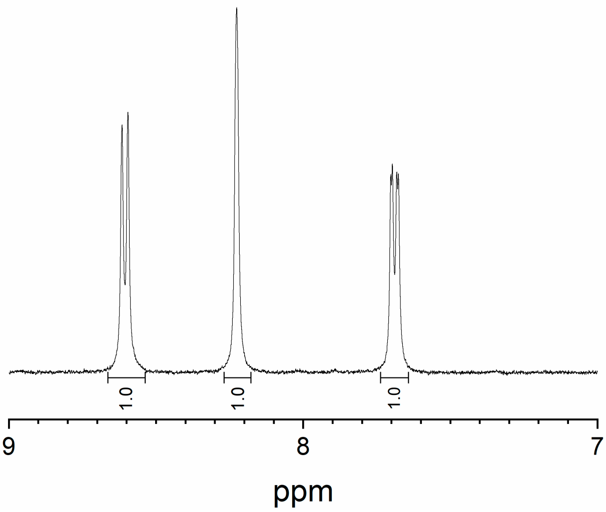

Figure 1. ^1H NMR spectrum of 4,4′-dimethyl-2,2′-bipyridine (bpy) in D_2O.

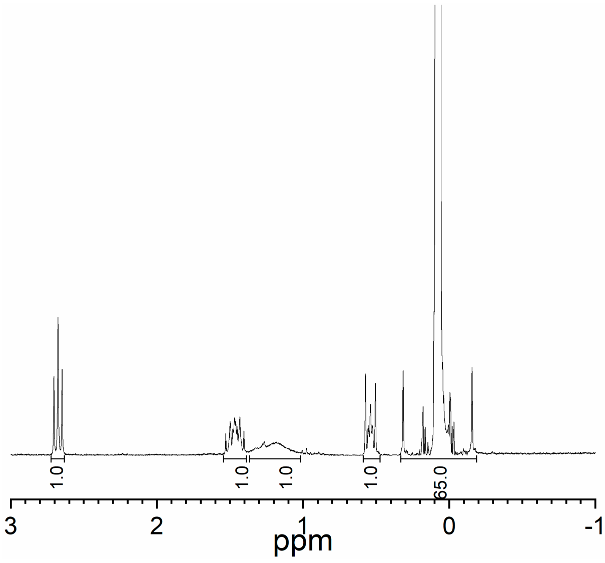

Figure S2. ¹H NMR spectrum of aminopropyl terminated poly(dimethylsiloxane) (PDMS) in $CDCl_3$.

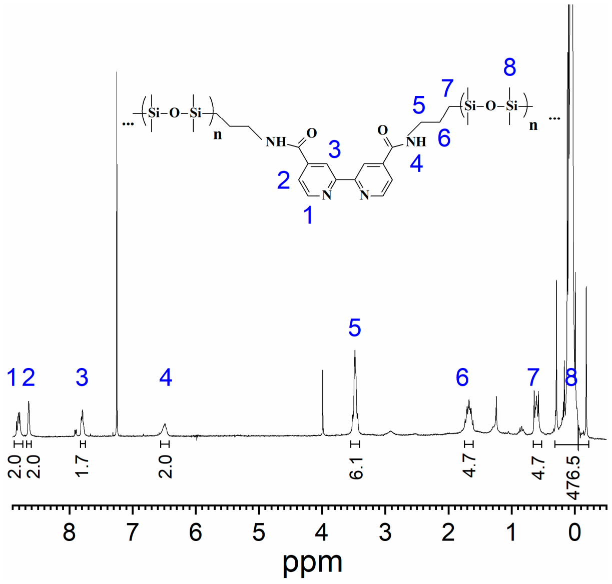

Figure S3. ¹H NMR spectrum of 2,2′-bipyridine-terminated poly(dimethylsiloxane)(bpyPDMS) in $CDCl_3$.

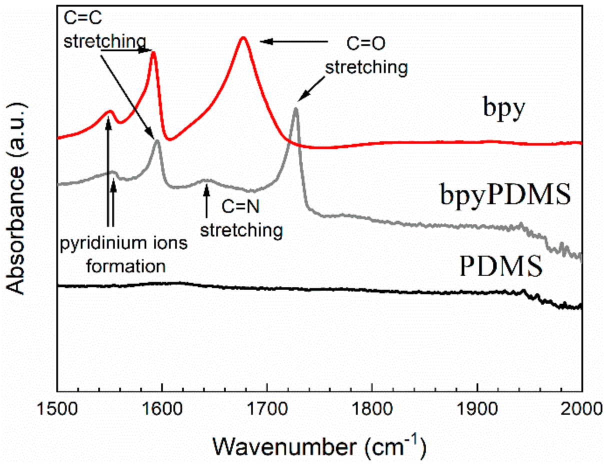

Figure S4. FT-IR spectra of bpy, bpyPDMS, and PDMS in the range of 1500–2000 cm^{-1}.

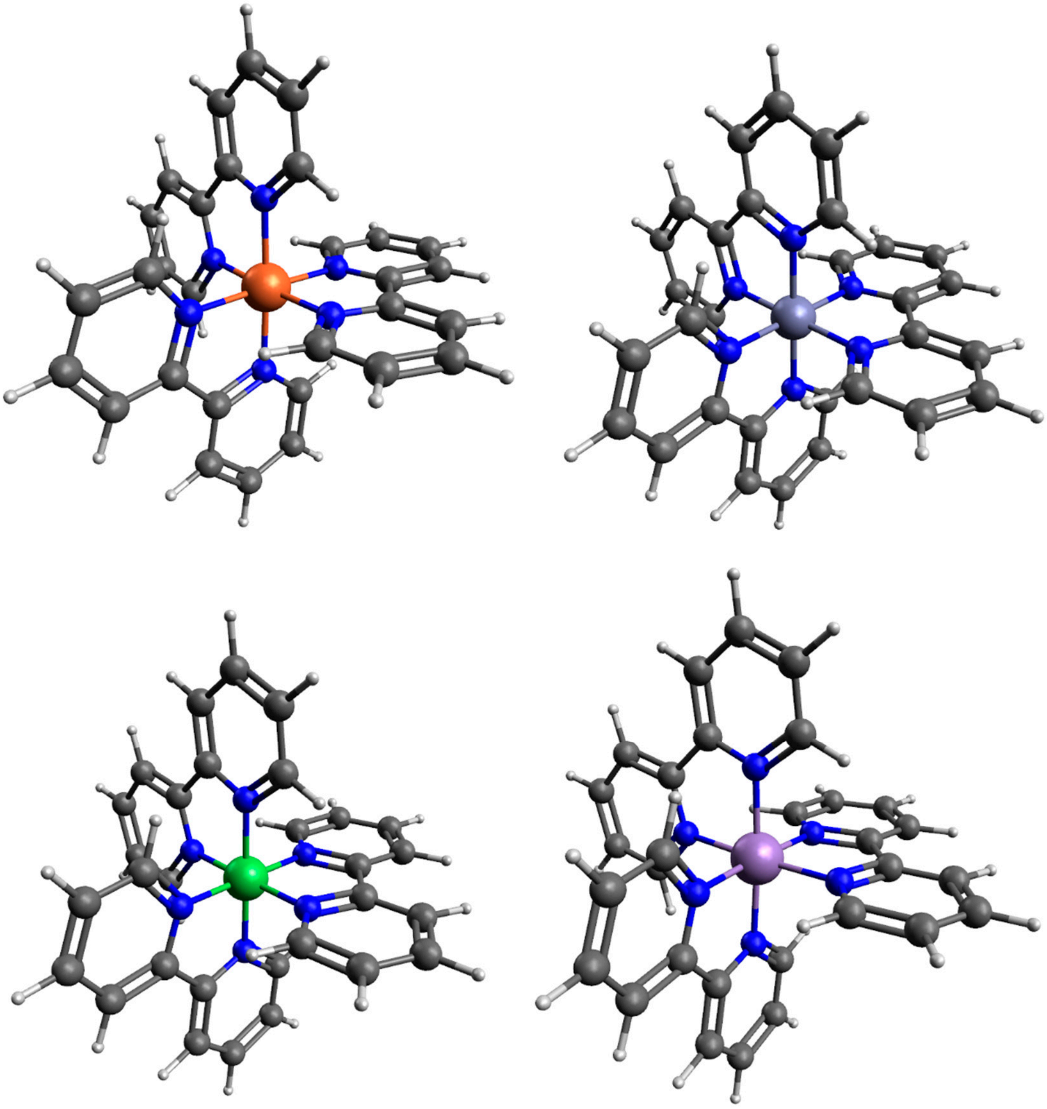

Figure S5. Geometry of optimized coordination compounds (Me: Mn^{2+}, Fe^{2+}, Ni^{2+}, Zn^{2+}).

Table S1. Geometry-optimized structure of 2,2′-bipyridine complexes.

$[Fe(bpy)_3]^{2+}$	$[Zn(bpy)_3]^{2+}$	$[Ni(bpy)_3]^{2+}$	$[Mn(bpy)_3]^{2+}$
$r_{Me\text{-}N}$ [Å]			
1.9541	2.2602	2.1417	2.3121
1.9549	2.2601	2.1409	2.3127
1.9548	2.2601	2.1401	2.3127
1.9544	2.2596	2.1411	2.3110
1.9538	2.2581	2.1405	2.3101
1.9546	2.2583	2.1401	2.3103
∢N-Me-N [°]			
81.85	73.06	77.04	71.72
95.30	97.93	97.26	98.69
86.16	78.06	82.10	76.47
87.57	97.85	97.28	98.50
95.32	91.76	88.93	92.49
87.51	91.73	88.99	92.42
95.42	97.88	97.23	98.64
95.28	78.00	82.11	76.32
86.10	97.91	97.26	98.67
95.45	73.59	77.03	71.71
95.40	97.88	97.22	98.64
87.53	77.94	82.07	76.27
81.87	91.74	88.93	92.49
81.84	97.81	97.26	98.47
86.26	73.56	76.99	71.68

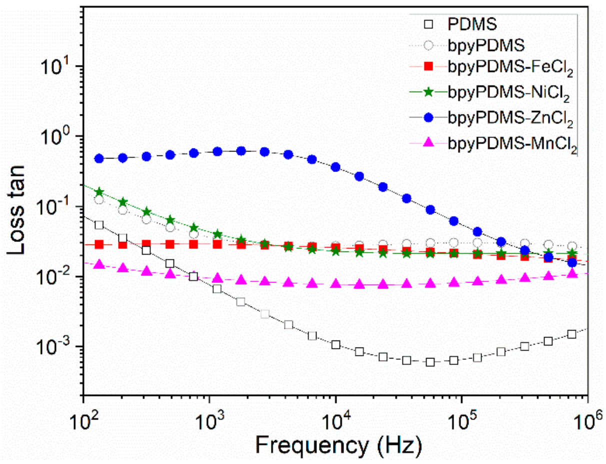

Figure S6. Frequency dependence of loss tangent at 293 K of PDMS and its metalloorganic complexes.

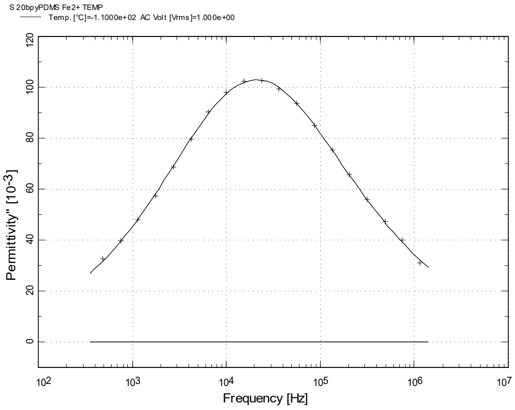

Figure S7. Exemplary of fitting procedure using WinFit software for bpyPDMS-$FeCl_2$ metalloorganic complex.
